# Identifying placebo responders and predictors of response in osteoarthritis: a protocol for individual patient data meta-analysis

**DOI:** 10.1186/s13643-016-0362-x

**Published:** 2016-10-28

**Authors:** Yu Fu, Monica S. M. Persson, Archan Bhattacharya, Siew-Li Goh, Joanne Stocks, Marienke van Middelkoop, Sita M. A. Bierma-Zeinstra, David Walsh, Michael Doherty, Weiya Zhang

**Affiliations:** 1 School of Healthcare, University of Leeds, Leeds, UK; 2 Academic Rheumatology, University of Nottingham, Clinical Sciences Building, City Hospital, Nottingham, NG5 1PB UK; 3 Arthritis Research UK Pain Centre, Nottingham, UK; 4 Arthritis Research UK Centre for Sports, Exercise and Osteoarthritis, Nottingham, UK; 5 Academic Orthopaedics, Trauma and Sports Medicine, Queens Medical Centre, University of Nottingham, Nottingham, UK; 6 Sports Medicine Unit, University of Malaya, Kuala Lumpur, Malaysia; 7 Department of General Practice, Erasmus MC Medical University, Rotterdam, The Netherlands

**Keywords:** Osteoarthritis, Placebo response, Individual patient data meta-analysis

## Abstract

**Background:**

The management of osteoarthritis (OA) is unsatisfactory, as most treatments are not clinically effective over placebo and most drugs have considerable side effects. On average, 75 % of the analgesic effect from OA treatments in clinical trials can be attributed to a placebo response, and this response varies greatly from patient to patient. This individual patient data (IPD) meta-analysis aims to identify placebo responders and the potential determinants of the placebo response in OA.

**Methods:**

This study is undertaken in conjunction with the OA Trial Bank, an ongoing international consortium aiming to collect IPD from randomised controlled trials (RCTs) for all treatments of OA. RCTs for each treatment of OA have been systematically searched for, and authors of the relevant trials have been contacted to request the IPD. We will use the IPD of placebo-controlled RCTs held by the OA Trial Bank for this project. The IPD in placebo groups will be used to investigate the placebo response according to the minimum clinically important difference (MCID) threshold (e.g. 20 % pain reduction). Responders to placebo will be compared with non-responders to identify predictors of response. The quality of the trials will be assessed and potential determinants will be examined using multilevel logistic regression analyses.

**Discussion:**

This study explores the varying magnitude of the placebo response and the proportion of participants that experience a clinically important placebo effect in OA RCTs. Potential determinants of the placebo response will also be investigated. These determinants may be useful for future studies as it may allow participants to be stratified into groups based on their likely response to placebo. The results of this study may also be useful for pharmaceutical companies, who could improve the design of their studies in order to separate the specific treatment from the non-specific contextual (i.e. placebo) effects.

**Systematic review registration:**

PROSPERO CRD42016033212

**Electronic supplementary material:**

The online version of this article (doi:10.1186/s13643-016-0362-x) contains supplementary material, which is available to authorized users.

## Background

Osteoarthritis (OA) is the most common form of arthritis worldwide [1]. People with OA often experience pain alongside impaired mobility and participation, resulting in reduced quality of life [2]. There is no cure for OA, and available treatments aim to optimise pain management using both pharmacological and non-pharmacologic modalities [3]. Of 51 currently available treatment options for the symptomatic management of OA, most do not achieve a minimum clinically important difference (MCID) (i.e. an effect size ≥0.5) over placebo [4, 5]. Furthermore, most pharmacological treatments have a number of side effects.

In contrast, research and clinical evidence show that placebo or sham interventions appear to be effective for symptom control in OA [6] and in other conditions including pain [7], depression [8], chronic fatigue syndrome [9], asthma [10], hypertension [11], and Parkinson’s disease [12]. Previous studies suggest that the benefits of placebo interventions are mainly for patient symptoms and distress, which are considered to be the principal treatment targets in people with OA [6]. A recent meta-analysis showed that, on average, 75 % of the analgesic effect from OA treatments can be attributed to placebo response and this response varies greatly from patient to patient [13].

However, debate continues as to whether the estimation of placebo response is adequate and accurate. This is largely due to the fact that the placebo response is measured as the overall change from baseline in the placebo group [14]. It is difficult to differentiate this from the regression to the mean and the natural disease fluctuation unless a no-treatment group or waiting-list group is included in the trial [15–17]. However, Vase et al. [18] argued that the placebo response is robust and enhances the treatment effect when conditions are amenable to placebo, when placebo is given as an analgesic or when participants are properly blinded [19]. Furthermore, a systematic review [14] involving 198 randomised controlled trials (RCTs) also suggested that placebo is effective at relieving pain and improving function and stiffness. Its effect size (ES = 0.51, 95 % CI 0.46 to 0.55) was found to be significantly greater than that observed in untreated, observation-only controls (ES = 0.03, 95 % CI −0.13 to 0.18).

When studying the placebo response, it is essential to employ correct and robust methodology. Given the fact that predictors of placebo response may be person-specific (e.g. age and gender) or study-specific (e.g. sample size and allocation concealment), we need to consider both person and study level characteristics. Individual patient data (IPD) meta-analysis permits both individual patient level and study level predictors of placebo response to be taken into account. Moreover, it allows the researchers to use the existing datasets from RCTs instead of undertaking a large and expensive trial. This approach has been advocated by both the European League Against Rheumatism (EULAR) and the Osteoarthritis Research Society International (OARSI), who have further endorsed an international collaboration for the development of OA Trial Bank [20]. The OA Trial Bank is an ongoing international consortium aiming to collect IPD from existing RCTs for all treatments in OA. Currently, the Bank has completed an IPD analysis for intra-articular (IA) glucocorticoids [21] and is collecting the IPD for glucosamine, topical non-steroidal anti-inflammatory drugs (NSAIDs), and topical capsaicin in OA. This provides a representative sample of the treatments in OA, spanning across various modes of delivery including tablets, injections, and topical formulations, for this project. The aim of this review is to identify placebo responders and predictors of response in OA.

## Methods/design

An IPD meta-analysis of RCTs will be undertaken to identify placebo responders and to investigate the predictors of placebo response in participants with OA. In order to increase transparency, the PRISMA-P checklist [22] was referred to as Additional file 1.

### Study selection

RCTs of IA glucocorticoids, glucosamine, topical NSAIDs, and topical capsaicin in OA have been systematically searched. Placebo-controlled RCTs collected in our IA glucocorticoids [21], glucosamine, topical NSAID, and topical capsaicin (PROSPERO registration number: CRD42016035254) [23] studies will be included in this IPD analysis.

#### Type of studies

All placebo-controlled RCTs identified for the above four treatments, including crossover trials, will be included. Trials for other types of arthritis, such as rheumatoid arthritis, will be excluded. Studies of non-clinical outcomes, including biomarkers and animal models, will be excluded. No language restrictions will be applied to this review.

#### Participants

Participants in the above RCTs diagnosed with OA, as defined by the criteria endorsed by the American College of Rheumatology [24, 25] or by the use of clearly defined radiographic and clinical criteria, will be included.

#### Types of interventions

This review focuses on the interventions used in the control arms of trials collected in the OA Trial Bank. This includes participants receiving placebo interventions (regardless of the mode of delivery and dose), participants on waiting lists, and participants under standard care.

#### Types of baseline assessments

As a minimum, studies will need to record pain, age, and gender at baseline. Other clinical characteristics, including physical function, stiffness, and pain elsewhere, will be included if available.

#### Types of outcomes

The minimum criterion for inclusion is reporting of pain. The primary outcome measure for investigating placebo response is pain reduction at 4 weeks of follow-up. Secondary analyses will include pain reduction at other durations of follow-up, as well as functional impairment and patient global assessment, as recommended by the OMERACT-OARSI Initiative [26, 27].

### Identification of eligible studies

Trials eligible for inclusion in this analysis will be identified from the IPD collected in the OA Trial Bank for IA glucocorticoids [21], glucosamine, topical NSAIDs, and topical capsaicin reviews (PROSPERO registration number: CRD42016035254) [23]. Each treatment was systematically reviewed (MM for IA steroid, JR for glucosamine, and MSMP for topical NSAIDs and capsaicin). The process was similar for all reviews and is summarised below.

A search strategy was developed by the lead reviewer and refined in consultation with team members, including physicians, librarians, and researchers experienced in conducting systematic reviews. A systematic literature search was then undertaken by the lead reviewer. Databases searched include Cochrane library, MEDLINE, Embase, AMED, Web of Science, Scopus, Pedro, and CINAHL [21]. The identified studies were exported to Endnote, where eligibility for inclusion was assessed by two independent reviewers. If no consensus was reached, a third researcher was consulted.

In addition to the systematic literature search, efforts were made to identify unpublished trials by contacting pharmaceutical suppliers and reviewing the British National Formulary, the electronic Medicines Compendium, and Clinicaltrials.gov.

### Data collection and transfer

All corresponding authors of eligible trials have been invited to collaborate by MM, JR, and MSMP. Corresponding authors that are interested in collaboration have been asked to sign a data delivery agreement that has been drafted on behalf of the OA Trial Bank. The agreement includes items on input data, obligations, ownership of data, terms, authorship, and publications. Data has been contributed to the OA Trial Bank by the corresponding author and is stored on a secure server. Data has been accepted in any format and is kept anonymised and confidential.

The corresponding authors of placebo-controlled trials stored in the OA Trial Bank will be contacted. The authors will be asked to sign a further data transfer agreement allowing the use of their data for this review before the transfer of the data to The University of Nottingham for analysis.

### Risk assessment

The quality of the included trials will be independently assessed by two reviewers using a modified version of the Risk of Bias tool recommended by the Cochrane Collaboration [28]. Whenever there is a disagreement, a third independent researcher will be consulted until a consensus is reached. The modified Risk of Bias Tool is composed of nine questions that measure each of the domains included in the tool (Table 1). For each domain, studies will be rated as a “low risk”, “high risk”, or “unclear risk” of bias. Clear criteria for the ratings will be followed, and a justification for the rating will be required. Studies will then be categorised as “low risk”, “high risk”, or “unclear risk”. A study with low risk is defined as fulfilling at least five of the criteria questions.

**Table 1:** Modified risk of bias assessment

	Yes	No	Unclear	Comments
1. Was the randomisation procedure adequate?	☐	☐	☐	
2. Was the treatment allocation adequately concealed?	☐	☐	☐	
3. Were participants blinded to the intervention?	☐	☐	☐	
4. Were physicians blinded to the intervention?	☐	☐	☐	
5. Were outcome assessors blinded to the intervention?	☐	☐	☐	
6. Incomplete outcome data: Is the attrition rate <15 %?	☐	☐	☐	
7. Are all pre-specified outcomes of interest reported in the pre-specified way?	☐	☐	☐	
8. Was intention-to-treat analysis used?	☐	☐	☐	
9. Were the treatment and control group similar at baseline?	☐	☐	☐	

All trials, irrespective of quality, will be included in the primary data analyses. Subsequently, a subgroup analysis will be undertaken to examine whether the quality of the study influences the outcomes, for example, whether higher quality studies have a greater placebo response.

### Data extraction

Data retrieved from the OA Trial Bank will include characteristics relating to the participant (age, gender, body mass index (BMI)); disease (radiographic information, signs of inflammation, muscle strength, duration of complaints, pain severity, type of pain, central sensitization, psychological assessments); placebo (topical/oral/injection, dose); trial (sample size, setting, allocation concealment, risk of bias); and outcome measures of interest (pain, function, patient global assessment, quality of life). All randomised participants with a database record will be entered into a pooled database, and all individual trials will be assigned an individual trial number.

### Outcomes

The primary dichotomous outcome for all analyses will be clinically important pain relief (present/absent) at 4 weeks after treatment. This is assessed using the visual analogue scale (VAS) pain score from 0 (no pain) to 100 mm (worst pain ever).

Clinically important pain relief will be defined as a 20 % or more reduction in VAS score from baseline within the placebo group. This threshold was chosen as it is the most commonly used threshold for the minimum clinically important improvement of relative change [29]. It has been suggested for pain and function for rheumatic diseases, including OA, and relies on the assumption that the waiting-list or observational group has no pain reduction [14]. In trials with a waiting-list group, significant pain relief is defined as a reduction in pain 12.5 % greater than that observed in the waiting-list group [30]. VAS pain score (continuous variable) will be used for secondary analyses.

If the VAS pain score has not been measured at 4 weeks, the time-point closest will be used instead. Pain scores at all other time points will be considered in the secondary analyses.

Where possible, the VAS pain score will be used for analysis. If unavailable, the WOMAC pain score will be used instead, followed by other Likert scores converted into a VAS 0–100 scale [21]. Other outcomes such as function (standardised to a 0–100 scale), global assessment, and quality of life will also be considered if available [26].

### Data analyses

Descriptive analyses will be performed to present the characteristics of each individual trial and the study participants. Means and standard deviations will be used to describe normally distributed continuous data whilst medians and interquartile ranges will be used for data that are not normally distributed. Categorical data will be described using frequencies and percentages. Statistical analyses will be performed using the statistical programme Stata SE 14 (StataCorp, College Station, TX).

Any missing data will be assumed to be missing at random (MAR); therefore, observed participant characteristics will be used to impute missing data using multiple imputation [31, 32]. Missing data will be imputed within each original study prior to IPD being pooled. To test the validity of the imputation, a sensitivity analysis will be carried out to compare the results from the complete dataset with those from the imputed dataset.

A one-step approach will be used as it has been demonstrated to have greater power and flexibility over the two-step approach [33]. A multilevel logistic regression model will be fitted, taking into account the hierarchical nature of the data. Significant pain relief will act as the dependent variable and will be used to estimate the magnitude of the placebo response. Random intercepts may be introduced in the model to account for the patients nested within each trial.

All characteristics listed in the section “Data Extraction” (participant; disease-specific; placebo-specific; trial) will be considered potential predictors. These have been chosen as the current literature suggests that they are related to pain or response to therapy in OA. Participant-related information, such as age, gender, and BMI will be used at the patient level. Trial characteristics will be used as covariates at the study level. In the secondary analysis, a time-point level will be introduced for time points other than 4 weeks.

According to the availability of a waiting-list/no-treatment control, two models will be developed. Where a waiting list is unavailable, a multilevel logistic regression model will be built. The dependent variable of the model will be placebo response (yes/no), and independent variables will include all potential predictors. In terms of the modelling strategy, we will first include all potential predictors in the model. We will then develop a model only including significant predictors (*p* ≤ 0.05). Finally, we will refine the model by re-introducing the previously excluded non-significant predictors, one by one, to examine their significance and influence on the model.

Where a waiting list is available, predictors will be identified by assessing the treatment-covariate interaction in the multilevel regression model. For this model, the dependent variable will still be the placebo response (yes/no). However, the independent variables will include both the treatment (placebo yes/no) and predictors. We will develop a model using one predictor at a time, the treatment variable (i.e. placebo yes/no) and the treatment-predictor interaction term. Other predictors will be adjusted for as covariates in the model [32, 33]. A significant predictor will be identified when the treatment-predictor interaction term is statistically significant (*p* ≤ 0.05) in the model. The overall estimate of the effect of the predictor, with a 95 % confidence interval and *p* value, will be presented.

In addition, secondary analyses will be conducted for VAS pain scores (continuous) using multilevel linear regression models. These will be conducted as detailed above, but the dependent variable will be replaced by VAS score and ANCOVA (analysis of covariance) will be used instead of the logistic regression.

The heterogeneity between trials will be tested using the *I* ^2^ value [34] and will be presented along with the 95 % confidence interval around it [35].

The potential for publication bias and small study effects will be examined using a contour-enhanced funnel plot and a statistical test for asymmetry [36, 37]. A *p* value less than 0.10 will be taken to indicate statistical evidence of asymmetry.

## Discussion

This review aims to provide evidence of how the placebo effect produces a clinically important improvement in patient-centred outcomes in RCTs of OA. Potential determinants of the placebo response in people with OA will be investigated, which may be useful for future studies. Defining the patient, disease, treatment, and delivery of care-specific factors that influences the placebo response may allow stratification of patients who are most likely to improve with placebo. As placebo effect is an integral part of the treatment effect 13, the results of this study will be useful to enhance the overall treatment effect. This study will also help refine the effect size of placebo interventions for pain reduction in OA, as current evidence has not allowed a consensus on the minimum perceptible clinical improvement for placebo interventions in OA [5, 29, 38].

Limitations of this review include the fact that the placebo responders are measured by the overall difference between baseline and endpoint, rather than the difference in effect between placebo and non-treatment groups. Future studies may be needed to examine the magnitude of the placebo response by comparing changes observed in participants in placebo groups with those not receiving treatment. As is often a limitation of IPD meta-analyses, it is possible that original trial authors may not be willing to collaborate or may not have access to the raw datasets required [39, 40]. Consequently, we may be underpowered and miss some significant predictors. However, several approaches for overcoming these challenges will be implemented when collecting data for the OA Trial Bank. For example, attempts will be made to obtain IPD from trials in the grey literature as well as directly from pharmaceutical companies. We will also facilitate the process of data collection for original authors by accepting the data in any format [40, 41]. Finally, conducting this review within a collaborative group like the OA Trial Bank allows pooling of resources which aims to facilitate the process [39].

The results of this study may have implications relevant to both clinical practice and pharmaceutical companies. Clinicians will be made aware of the importance of the placebo response and its determinants. This may enable them to develop individualised treatment plans for patients that maximise the overall treatment effect, thereby improving symptoms and increasing patients’ satisfaction. The results of this study may also be of interest to pharmaceutical companies who could recruit participants who are likely to have a low placebo response in their trials. This would increase the likelihood of observing a benefit for their drug over and above placebo.

## Additional file

Additional file 1: PRISMA-P (Preferred Reporting Items for Systematic review and Meta-Analysis Protocols) 2015 checklist: recommended items to address in a systematic review protocol*. (DOC 895 kb)

## Abbreviations

AMED: The Allied and Complementary Medicine Database
ANCOVA: Analysis of covariance
BMI: Body mass index
CINAHL: Cumulative Index to Nursing and Allied Health Literature
ES: Effect size
EULAR: European League Against Rheumatism
IA: Intra-articular
IPD: Individual patient data
MAR: Missing at random
MCID: Minimum clinically important difference
NSAID: Non-steroidal anti-inflammatory drug
OA: Osteoarthritis
OARSI: Osteoarthritis Research Society International
OMERACT: Outcome Measures in Rheumatology
PRISMA-P: Preferred Reporting Items for Systematic review and Meta-Analysis Protocols
PROSPERO: International Prospective Register of Systematic Reviews
RCTs: Randomised controlled trials
VAS: Visual analogue scale
WOMAC: Western Ontario and McMaster Universities Pain Scale
